# CONSUMERS AND PROVIDERS ON THE NEEDS OF LONG-TERM SURVIVORS AND PEOPLE AGING WITH HIV IN NEW YORK STATE

**DOI:** 10.1093/geroni/igac059.651

**Published:** 2022-12-20

**Authors:** Maria Brown, Eugenia Siegler, Marz Albarran, John Wikiera, Angie Partap, Courtney Ahmed, Sheriden Beard, Thomas Heslop

**Affiliations:** Syracuse University, Syracuse, New York, United States; Weill Cornell Medical College, New York, New York, United States; HIVStopsWithMe.Org, Renssaelaer, New York, United States; NY Statewide Peer Network, Rome, New York, United States; Stony Brook Medicine, Patchogue, New York, United States; NYSDOH AIDS Institute, Brooklyn, New York, United States; NYSDOH AIDS Institute, New York, New York, United States; Weill Cornell Medical College, Washgton, District of Columbia, United States

## Abstract

**Objectives:**

To document the practical needs, and develop quality initiatives to address those needs, of the growing population of long-term survivors (LTS) and older people with HIV (OPH) in New York State.

**Methods:**

The HIV+ Aging/LTS/Perinatally Diagnosed Subcommittee of the NYS Quality of Care Consumer Advisory Council used community based participatory research methods to design a statewide survey based on categories identified in August 2020 virtual town halls with consumers and providers across New York. Syracuse University launched the survey, open to consumers aged 18 and over who were LTS or OPH, clinicians, and social services providers, in June 2021 using Qualtrics™. Participants chose the three most important barriers and recommendations for each category. Responses were characterized using basic descriptive statistics.

**Results:**

Participants included 124 consumers from 26 counties, 20 clinicians, and 24 social service providers. Two thirds of participants were cisgender men (67%), 27% were African American, 80% identified as both LTS and OPH. On average, consumers were 58 years old, had been living with their HIV+ diagnosis for 27 years, and reported 4 additional conditions, most commonly depression (30%). LTS and OPH were concerned about clinical and financial needs, particularly coordination of clinical care, unmet housing needs, cultural representation in mental health services, and financial support of LTS and OPH. Implications: Community based participatory research can inform and stimulate changes in clinical care for LTS and OPH. Survey results are informing a plan for functional screening of OPH and LTS that can be performed by certified peer workers.

